# Our Hospitals and Christmas Day

**Published:** 1911-12-23

**Authors:** 


### The Hospital

The Workers' Newspaper of Administrative Medicine and Institutional Life, Administration, National Insurance and Health.

No. 1328, Vol. LI. SATURDAY, DECEMBER 23RD, 1911. PRICE ONE PENNY.

### OUR HOSPITALS AND CHRISTMAS DAY.

Every undertaking of importance has at least one day in the year when every person responsible for its affairs must be on duty. If the undertaking be a vast enterprise or a great business the day of the year is usually that on which the shareholders assemble, when the responsible managers have to give an account of their stewardship. Without these annual stock-takings and an overhaul of all the departments efficiency would tend to diminish, and results might properly be expected to become less and less satisfactory year by year. Some readers may inquire what has this to do with the voluntary hospital, which is anything but a business? Our answer is plain. Never before in the history of the world has the business of charity been so generally recognised, or so absolutely important, as it is to-day. We could never have had the modern voluntary hospital alert, efficient, embracing every portion of the scientific field, and fruitful in results for good to an ever-increasing extent, unless hospital managers had recognised that to succeed the voluntary hospital, in all its ramifications, must be treated as a great business organisation.

It is popularly but erroneously supposed that there were no hospitals before Christ. That is not the fact. The hospital idea took shape long before Christ, but the hospital at its best, as we know it to-day, only became possible and has continually developed from the day on which the Sermon on the Mount was preached. No one who has not entered personally into the daily life of a modern voluntary hospital, who has missed the attraction and inspiration which such a life offers to the best of human kind, who has failed absolutely to lose self in whole-hearted service for others, can appreciate its commanding influence for good and the abiding love which it inspires in the real worker who has lived such a life from day to day. We have often said, and we would give much to secure, that every man and woman, with any brain power worthy of the name, should spend at least one Christmas Day in a great voluntary hospital. It is increasingly the fact—owing, as we believe, to the enormous growth in the mania for what is called amusement and continuous exercise which possesses the present generation—that self is becoming more and more a fetish in the individual, to his and her undoing and moral injury. Yet so strongly does the influence of hospital life make for good in the individual that its power is sufficient to annihilate self, so far as hospital workers are concerned, whether they be students or sisters and nurses, resident officers or those supporters of such institutions, who have come within the circle of knowledge.

Christmas Day proves this to demonstration. You enter the hospital and you find that the whole staff and each group of workers we have just named, with many others, are on duty, absorbed in the wholesome endeavour to make Christmas in the hospital a memorable day to the suffering and the sick. Everybody everywhere has forgotten their individuality. For all, whether they know it or not, are inspired with the true Christian spirit, and their one thought is for the patients, the poor sufferers in the beds, how best to make them feel at home. The worker's wish to enable them to realise and understand that patients individually and collectively are the whole-hearted concern of the owner of every bright face which surrounds them and ministers to their happiness and cheer from sunrise to sunset. Words fail us to describe the best of our voluntary hospitals on Christmas Day, but we can say with truth that there is nowhere else in the world where a good man or woman can reap greater benefit or renew their strength for the great battle of life to an equal degree to that they may happily garner within these hospital walls. Surely facts like these are the best and strongest of evidence that institutions directly traceable to the Sermon on the Mount have resulted, not only in permanent good to the suffering and the sick, but in a spiritual gain to succeeding generations of people, who, as individuals, may have never entered a hospital, who have certainly never been a patient within its walls. We should like to thank all those who render personal service in our hospitals on Christmas Day from one end of the Empire to the other. Only we know, as old workers ourselves, that thanks cannot be given and ought not to be given, where the joy and the privilege of service is so gladsome a thing in itself, and so charged with good and blessedness to the workers.

The Hon. John Griffiths, in his speech at St. James's Palace to the League of Mercy, published elsewhere, has given all workers a foreword and inspiration, not only for this season, but for the year which is to follow. In a graphic illustration, he mentions that General Harrison, speaking of an incident in the American Civil War, declared that the commander of each regiment, owing to the nature of the country traversed, could not for days and days see more than half of his own line, whilst the lines to the right and left were wholly hidden. That description is typical of much of the work which most workers have to do day by day throughout each year in a great hospital. Each has to go forward, responsible for certain special work, dependent mainly upon themselves, but still one of a great army, all working side by side to secure the defeat of disease and death. So, like the army described by General Harrison, there comes a day —Christmas Day in the case of our great voluntary hospitals—when the whole army stands revealed and joins hands under one flag, the flag which was raised by Christ Himself on the Mount. It is well that we workers should take stock now and then, and especially at this season. It is, no doubt, good for the work and good for ourselves that we should have so much to do on most days of each year that we sink our individuality, and often fail to realise its existence in fact. But there is an inspiration, an encouragement, a power of concentration and judgment in work, the best of which would be unattainable if we workers did not sometimes join hands, moved by the same spirit. Thus we find ourselves standing shoulder to shoulder on Christmas Day, uniting our energies and forces to make Christ's poor, (with ourselves) realise the power of His life and the strength of His teachings for good, to all mankind.

